# Upconverting phosphor technology-based lateral flow assay for the rapid and sensitive detection of anti-*Trichinella spiralis* IgG antibodies in pig serum

**DOI:** 10.1186/s13071-021-04949-2

**Published:** 2021-09-22

**Authors:** Jian Li, Jing Ding, Xiao-Lei Liu, Bin Tang, Xue Bai, Yang Wang, Wei-Dong Qiao, Ming-Yuan Liu, Xue-Lin Wang

**Affiliations:** Key Laboratory of Zoonosis Research, Ministry of Education, Institute of Zoonosis, College of Veterinary Medicine, Jilin University, OIE Collaborating Center On Foodborne Parasites in the Asian-Pacific Region, Changchun, China

**Keywords:** *Trichinella spiralis*, Upconverting phosphor technology, Lateral flow assay, Excretory-secretory antigens, Serodiagnosis

## Abstract

**Background:**

*Trichinella spiralis* is a zoonotic food-borne parasite. A disease caused by infection with *T. spiralis* is called trichinellosis in humans. It is important to investigate the epidemic situation and the surveillance of herds and then prevent infection in humans. Therefore, this study is to develop a rapid and sensitive diagnostic method for on-site test in domestic and wild animals.

**Methods:**

Upconverting phosphor nanoparticles (UCNPs), an excellent optical label, were conjugated with the excretory-secretory (ES) antigens from *T. spiralis* muscle larvae (ML) or goat anti-rabbit IgG, and a lateral flow (LF) assay based on these probes (UCNPs-ES/goat anti-rabbit IgG) was developed for the rapid and sensitive detection of anti-*T. spiralis* IgG antibodies in pig serum. The assay is named the UPT-LF-ES assay. In addition, the probes were characterized, and the assay was optimized. A cut-off threshold of the assay was also identified by using 169 known negative pig samples. Performance of the assay to *T. spiralis* with different infective numbers, cross-reactivity with other parasitic infections, the single-blinded experiment, and coincidence were evaluated with the assay.

**Results:**

The UPT-LF-ES assay was successfully constructed and optimized based on the probes of UCNPs-ES/goat anti-rabbit IgG. In the pigs infected with 100, 1000, and 10,000 ML, positive results were first presented at 35 days post-infection (dpi), 30 dpi, and 25 dpi, respectively. The assay had no cross-reaction with other parasitic infections. A single-blinded experiment indicated that the sensitivity and specificity of the UPT-LF-ES assay were 100% and 100%, respectively, the area under the receiver operating characteristic (ROC) curve was 1.000. In addition, the value detected by the UPT-LF-ES assay was significantly different between positive and negative samples. Moreover, compared with the “gold standard” magnetic stirrer method, the coincidence rate of the UPT-LF-ES assay was 87.27%, and the kappa (K) coefficient was 0.7454, showing a substantial agreement.

**Conclusions:**

The UPT-LF-ES assay is a useful point-of-care test (POCT) with *T. spiralis* in the detection of pig, which contributes to preventing human trichinellosis.

**Graphical Abstract:**

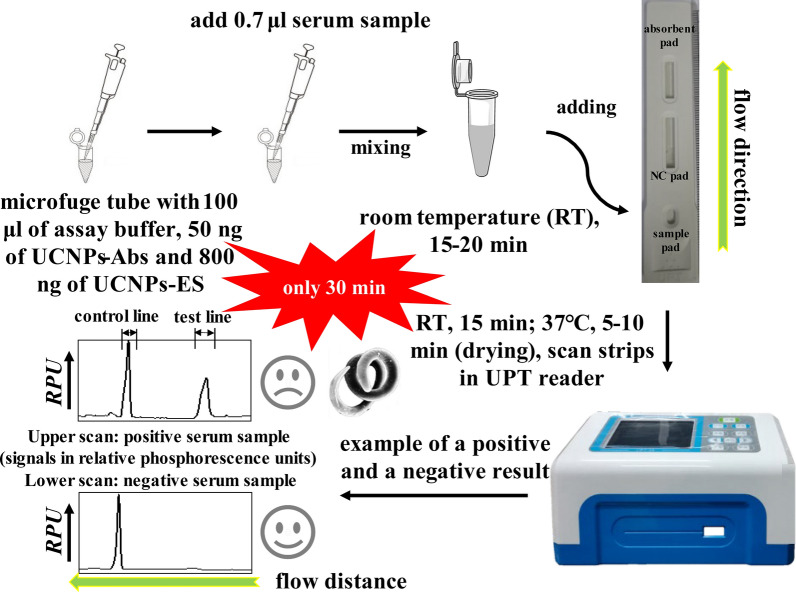

**Supplementary Information:**

The online version contains supplementary material available at 10.1186/s13071-021-04949-2.

## Background

*Trichinella* is a zoonotic food-borne parasite, and humans or animals are infected by eating raw or undercooked meat that contains the infective larvae of *Trichinella* nematodes [1–3]. The infection of humans is called trichinellosis. According to reports, over 2500 people are infected annually by *Trichinella* worldwide [4]. The International Commission on Trichinellosis (ICT) reported that approximately 65,818 humans were infected by *Trichinella* worldwide between 1986 to 2009 [5]. China is currently one of the countries with the highest infection rates [2]; more than 600 trichinellosis outbreaks have occurred and approximately 40,000 people were infected, with 336 deaths from 1964 to 2011 [6, 7]. *Trichinella spiralis* (*T. spiralis*) is the most important species in the genus *Trichinella* with respect to public health due to its considerably high prevalence and good adaptation in domestic and wild pigs. Therefore, diagnosis, and surveillance of *T. spiralis* infection in domestic and wild animals are highly significant to preventing trichinellosis in China.

At present, the standard method of diagnosis of trichinellosis or *T. spiralis* infection by the World Organization for Animal Health (OIE) is the artificial digestion method [8]. Furthermore, enzyme-linked immunosorbent assay (ELISA) is recommended as the serological detection method for *T. spiralis* infection by the ICT [3, 9, 10]. However, these methods are difficult to detect *T. spiralis* infection rapidly and require related experimental skills; therefore, a rapid and simple test demonstrating active *T. spiralis* infection would be worthwhile in the on-site test. In this research, a lateral flow (LF) test using pig serum was developed to detect the anti-*T. spiralis* IgG antibodies, which can be conveniently used as a point-of-care test (POCT).

LF tests are widely applied and include tests for pregnancy, infectious diseases, cardiovascular disease, cancer biomarkers, toxins, and foodborne pathogens [11–16]. LF test based on colloidal gold-labeled excretory-secretory (ES) antigens of muscle larvae (ML) has also been developed to detect *T. spiralis* infection [17, 18]. Compared with gold nanoparticles, the LF test using upconverting phosphor nanoparticles (UCNPs) is more sensitive (approximately tenfold) and robust in some aspects [19–22], due to the unique feature of using the lower energy 980 nm infrared light (excitation light) to generate higher energy visual light (emission light) [23]. This light process is called upconversion, which does not happen in biological nature. Thus, UCNPs as a reporter label do not generate background fluorescence (autofluorescence) compared with conventional fluorescent labels, such as fluorescently labeled nanoparticles and quantum dots. In addition, UCNPs do not fade [24], allowing the LF strips based on UCNPs to be stored in the long term [25]. More importantly, the LF test based on UCNPs has no interference with from red blood cell hemolysis that is a problem sometimes encountered in LF test based on colloidal gold-labeled using finger stick blood [26]. Moreover, upconverting phosphor technology (UPT) based on the LF test has been employed as a portable detection device that can achieve quantitative detection with *Yersinia pestis*, *Brucella* spp., and *Bacillus anthracis* spores [27]. However, there is no report on the applications of a UPT-LF test for *T. spiralis* infection in pigs.

**Fig. 1:**
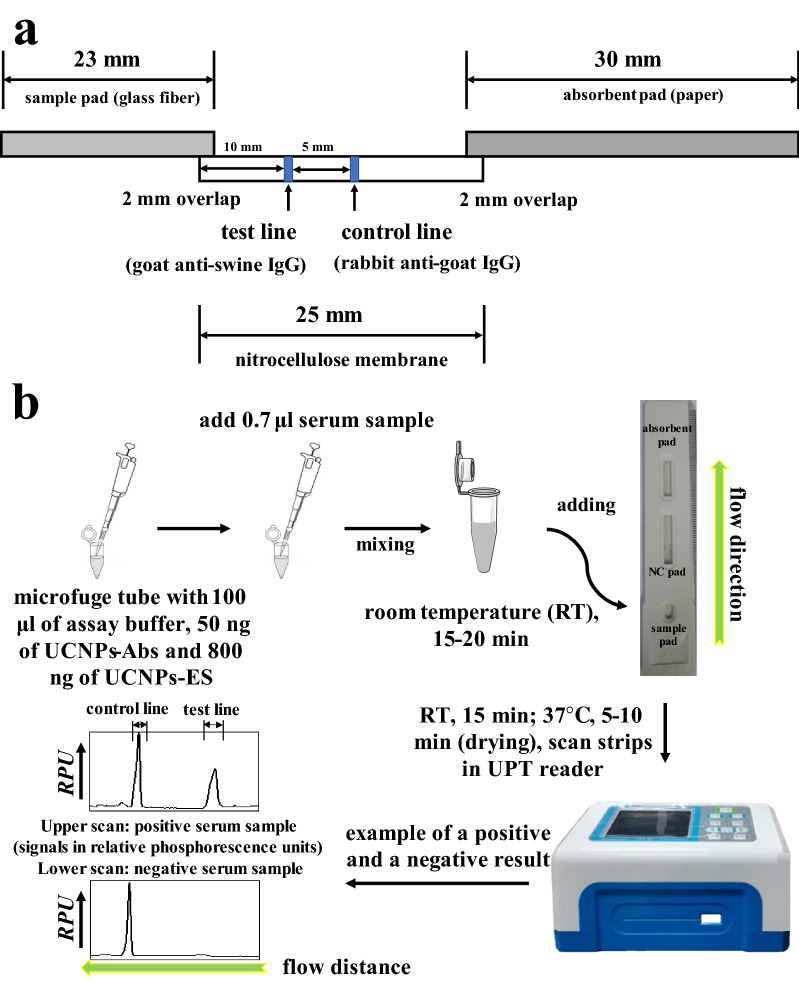
The UPT-LF-ES assay. **a** Schematic illustration of the UPT-LF-ES strip: test line 100 ng of goat anti-swine IgG and control line 100 ng of rabbit anti-goat IgG. **b** The LF protocol for antibody detection included four steps in series

For the first time, we designed an optimized UPT-LF-ES assay using pig serum samples to detect anti-*T. spiralis* IgG antibodies. A schematic diagram of the strip and operating procedures is shown in Fig. 1. Goat anti-swine IgG and rabbit anti-goat IgG were immobilized on nitrocellulose (NC) membranes as the test line (T-line) and control line (C-line), respectively. UCNPs labeled with ES (UCNPs-ES) and UCNPs labeled with goat anti-rabbit IgG (UCNPs-goat anti-rabbit IgG) were prepared in advance. Anti-ES IgG from the serum samples of pigs binds with UCNPs-ES first. Then, UCNPs-ES-anti-ES IgG is captured by goat anti-swine IgG coated on the T-line. Additionally, the UCNPs-goat anti-rabbit IgG is captured by the rabbit anti-goat IgG coated on the C-line. The results of the test are positively related to the phosphorescence intensity of the T-line. Furthermore, the sensitivity, specificity, and coincidence of the assay were evaluated, and the results showed satisfactory performance for the assay overall.

## Methods

### Reagents and materials

Sodium chloride (NaCl), Triton X-100, Tween 20, 2-amino-2-hydroxymethyl-propane-1,3-diol (Tris), sodium hydroxide (NaOH), glycine, bovine serum albumin (BSA), Coomassie blue dye solution, and 4-(2-hydroxyethyl) piperazine-1-ethanesulfonic acid (HEPES) were obtained from Beijing Solarbio Science & Technology Co., Ltd. (Beijing, China). Enhanced chemiluminescent (ECL) reagent was purchased from Thermo Fisher Scientific Inc. (Waltham, MA, USA). *N*-hydroxysulfosuccinimide sodium salt (sulfo-NHS), and 1-(3-dimethylaminopropyl)-3-ethylcarbodiimide (EDC) were purchased from Aladdin Company (Shanghai, China). Sodium azide (NaN_3_) was purchased from Sigma-Aldrich (St. Louis, MO, USA). Methanol and NaH_2_PO_4_·2H_2_O were purchased from Beijing Chemical Reagent Company (Beijing, China). Millipore Milli-Q water (> 18 MΩ cm) was used for all solutions. Horseradish peroxidase (HRP)-conjugated goat anti-swine IgG, goat anti-swine IgG, rabbit anti-goat IgG, and goat anti-rabbit IgG were purchased from Beijing Baiolaibo Technology Co., Ltd. (Beijing, China).

NH_2_-modified UCNPs (NaYF_4_: Yb^3+^, Er^3+^) were obtained from Shanghai Shunna Biotech Co. Ltd. (Shanghai, China). The excitation peak was 980 nm and that of emission was 545 nm. 0.45 μm polyvinylidene fluoride (PVDF) membrane was purchased from GE Healthcare (Chicago, IL, USA). Nitrocellulose membranes (Hi-Flow Plus HF135) and sample pads (SureWick glass fiber) were purchased from Millipore Corporation, and the absorbent pads and plastic backing were purchased from Shanghai Jieyi Biotechnology Co., Ltd. (Shanghai, China).

### Instruments

A multifunctional imaging system was purchased from Analytik Jena GmbH (Upland, CA, USA). A XYZ3060 Biostrip Dispenser and a CM 4000 Guillotine Cutter were purchased from BioDot (Irvine, CA, USA). A vortex mixer was obtained from Tiangen Biochemical Technology (Beijing) Co., Ltd. (Beijing, China). An Allegra™ X-22 centrifuge was obtained from Beckman Coulter Inc. (Carlsbad, CA, USA). An ultrasonic water bath (power 20 kHz, max power 320 W) was obtained from Dongguan Kangshijie Ultrasonic Technology Co., Ltd. (Dongguan, China). A transmission electron microscope (TEM, H-7650) was purchased from Hitachi (Tokyo, Japan). A fluorescence spectrometer system was purchased from Beijing ZOLIX Instruments Co. (Beijing, China). A 980 nm optical laser (Max 4 W) was obtained from Ningbo Yuanming Laser Technology Co., Ltd. (Ningbo, China). An upconverting phosphor technology-based biosensor (UPT-based biosensor) was obtained from the Shanghai Institute of Optics and Fine Mechanics, Chinese Academy of Science (Shanghai, China).

### Animals and parasites

Female Wistar rats weighing approximately 120 g were purchased from Norman Bethune University of Medical Science (NBUMS), China. *Trichinella spiralis* (ISS534) preserved in the Food-Borne Parasitology Laboratory of Key Laboratory for Zoonoses, Jilin University, was confirmed by the OIE Collaborating Center on Foodborne Parasites in the Asian Pacific Region and maintained by continuous passage infection in our laboratory.

### Preparation and characterization of ES antigens from ML (ML-ES)

Some previously reported methods were used to prepare ES antigens [17, 28]. Briefly, ten Wistar rats were inoculated with 3000 *T. spiralis* ML per rat by the oral route. The infected rats were euthanized 35 days post-infection (dpi), and the ML were recovered from the muscle tissues of rats with artificial digestion fluid (1% pepsin/HCl) [29]. After washing three times with 0.01 M phosphate-buffered saline (PBS, pH 7.2), the ML were resuspended in RPMI-1640 medium (Gibco BRL, Grand Island, NY, USA) containing antibiotics (100 U/ml penicillin, 100 μg/ml streptomycin) at approximately 5000 worms/ml and then incubated at 37 °C in an atmosphere containing 5% CO_2_ for 18 h. The ML were removed by filtration (0.22 μm filter, Millipore, USA) to obtain the filtrate containing ES antigens. The filtrate was concentrated using a 3 kDa ultrafilter (Millipore, USA), and then PBS was used to exchange the medium. The concentration of ES antigens from the ML was measured by the bicinchoninic acid method (BCA Kits, Beyotime Biotechnology, Shanghai, China). Moreover, the ES antigens were characterized by sodium dodecyl sulfate polyacrylamide gel electrophoresis (SDS–PAGE) and western blotting (WB). Briefly, 10 μg and 15 μg ES antigens were separately incubated in loading buffer at 100 °C for 10 min, and the mixed samples were subjected to SDS-PAGE on a 4% concentration gel and 12% separation gel. The separation gel was stained with Coomassie blue dye solution at room temperature (RT) for 2 h, and the gel was subsequently recorded using a camera. Meanwhile, the ES antigens in the paralleled separation gels were transferred to PVDF membranes. After blocking in TBST-B (25 mM Tris, pH 8.0, 125 mM NaCl, 0.05% Tween 20 (V/V), 3.7% BSA) at RT for 2 h, the membranes were incubated with the primary antibodies (10,000 T*. spiralis*-infected pig serum, 60 dpi or normal pig serum) at a dilution of 1:200 in TBST-B for 12 h at 4 °C. Secondary antibody (HRP-conjugated goat anti-swine IgG) at a dilution of 1:1000 in TBST-B was incubated with the membranes for 2 h at RT. The membranes were reacted with ECL reagent and exposed to a multifunctional imaging system. The ES antigens were stored at − 80 °C until use.

### Preparation and characterization of UCNPs-ES/goat anti-rabbit IgG

ES-COOH/goat anti-rabbit IgG-COOH was pre-activated to its succinimide by using EDC and sulfo-NHS and then reacted with an NH_2_-UCNP fragment [30]. Briefly, 1 ml of ES/goat anti-rabbit IgG solution (300 μg/ml in 10 mM NaH_2_PO_4_, pH 6.0) was incubated with 10 μl of 50 mg/ml EDC and 10 μl of 50 mg/ml sulfo-NHS overnight at RT with gentle shaking. Then, HEPES buffer (100 mM pH 7.4) was used to exchange the NaH_2_PO_4_ buffer of the reaction system with a 3 kDa ultrafilter. The resulting sulfo-NHS-activated ES/antibody was covalently linked to 500 μl of NH_2_-UCNPs (10 mg/ml in HEPES, pH 7.4) overnight at RT. After the conjugation reaction, the UCNPs-ES/goat anti-rabbit IgG was separated from free ES/goat anti-rabbit IgG by centrifugation at 28,500×*g* for 10 min. Next, the UCNPs-ES/goat anti-rabbit IgG was resuspended in UCP storage buffer (50 mM glycine, 0.03% Triton and 0.1% NaN_3_, pH 8.0) at 1 μg/μl and stored at 4 °C for up to 6 months. Morphological examination of UCNPs-ES/goat anti-rabbit IgG was performed by transmission electron microscopy (TEM), which was compared with unconjugated UCNPs, and from the sizes of the conjugated and unconjugated UCNPs were measured by software (Image-Pro Plus). Upconversion (UC) emission spectra of the conjugated and unconjugated UCNPs were recorded under 980 nm excitation light, and physical images were obtained in dark and light environments, respectively.

### Development and optimization of the UPT-LF-ES assay

The strip was composed of a sample pad, an NC membrane, an absorbing pad, and a plastic backing. A schematic illustration of the strip is shown in Fig. 1a. After the strip was assembled, the assay was optimized in the goat anti-swine IgG volume (T line) (400, 600, 800, 1000 ng/4 mm) and the sample dilution (1:100, 1:150, 1:200, 1:250, 1:300). According to the optimization results, the strip was prepared as follows: goat anti-swine IgG (800 ng/4 mm) and rabbit anti-goat IgG (100 ng/4 mm) in 10 mM Tris–HCl (pH 8.0) were dispensed as the T-line and C-line on the NC membrane, respectively. The membrane was dried at 37 °C for 2 h and then assembled on the plastic backing, which was positioned for a 2 mm overlap between the NC pad and the sample pad or absorbing pad. The assembled cards were cut into a strip 4 mm wide using a CM 4000 Guillotine Cutter. The finished strips were stored in a container with a dry pack at RT (expiration date: 6 months) until testing.

### UPT-LF-ES assay procedure

A schematic illustration of the assay is depicted in Fig. 1b. In detail, the assay included four steps. UCP storage buffer containing UCNPs-ES/goat anti-rabbit IgG was suspended by vortexing for 10 s. The desired amounts of UCNPs-ES (200 ng) and UCNPs-goat anti-rabbit IgG (50 ng) were diluted in UPT-LF-ES assay buffer (100 mM HEPES pH 7.5, 270 mM NaCl, 0.5% v/v Tween 20, 1% v/v BSA) to a final volume of 100 μl and sonicated to homogenize potential aggregates [26]. A 0.7 μl serum sample was added to the UPT-LF-ES assay buffer containing UCNPs-ES and UCNPs-goat anti-rabbit IgG and mixed. The mixed solution was incubated at RT for 15–20 min. The mixture was added to the sample pad of the UPT-LF-ES strip, which was placed for 15 min at RT and then for 5–10 min at 37 °C. The strip was scanned with a UPT-based biosensor. The results are expressed as a ratio signal (UPT value T/C).

### Cut-off threshold

To assess clinical specificity, the UPT-LF-ES assay was performed with 169 known negative pig sera samples obtained from the Food-Borne Parasitology Laboratory of Key Laboratory for Zoonoses, Jilin University.

### Serum samples

#### Serum samples from *T. spiralis* experimental infection

Nine Large White pigs (females, 2 months old, 20 kg) were obtained from the Experimental Animal Center of Norman Bethune University of Medical Science (NBUMS), China. Before the experiment, all pigs were tested using routine blood examination and fecal samples, and the results indicated that these pigs were healthy. Then the pigs were randomly divided into three groups and experimentally inoculated with 100, 1000, or 10,000 *T. spiralis* ML. Serum samples were collected and prepared on 0, 7, 9, 11, 13, 15, 17, 19, 21, 25, 30, 45, 60, 90, and 120 dpi, according to the collection method from this article [31]. The average larvae per gram (LPG) value was calculated and presented in a previously published article from our lab [31]. These serum samples were also tested in the UPT-LF-ES assay.

#### Serum samples with heterologous infections

A set of 8 samples from pigs infected with *Toxoplasma gondii* (*n* = 2), cysticerci of *Taenia solium* (*n* = 2), and cysticerci of *Taenia asiatica* (*n* = 2) was tested to evaluate potential cross-reactivity with other parasitic infections. These sera were provided by the Food-Borne Parasitology Laboratory of Key Laboratory for Zoonoses, Jilin University.

### Single-blinded experimental validation

Validation of the assay was performed using the sera from 35 positive samples that included different infections at 120 dpi, and these samples were randomly arranged between a set of 20 negative samples. These 35 positive serum samples were randomly selected in the serum sample bank of our laboratory [31]. These 55 serum samples were confirmed using the magnetic stirrer method and WB by the Food-Borne Parasitology Laboratory of Key Laboratory for Zoonoses, Jilin University, and were tested with the assay in a single-blinded experiment that was performed by a common tester who did not participate in this assay’s early development. The results from this experiment were evaluated by Student’s *t*-test and receiver operating characteristic curve (ROC curve) analysis.

### Data analysis

Peak areas for the T- and C-lines were calculated as relative intensity units by software from the Shanghai Institute of Optics and Fine Mechanics, Chinese Academy of Science, and the ratio values at which the T-line signals were divided by the C-line signals of each strip. Data were entered into Origin 2020b and are presented as histograms. The kappa (*K*) coefficient was employed to measure agreement between two methods, which allowed us to measure agreement beyond what was expected by chance alone [32, 33]. The generic formula for *K* is

$$K = \, (P_{o} - P_{e} )/\left( {1 - P_{e} } \right),$$

where *P*_*o*_ and *P*_*e*_ are the observed and expected proportions of agreement [34]. *K* > 0.8 and ≤ 1.0 show an almost perfect agreement between two methods, and *K* > 0.61 and ≤ 0.8 show a substantial agreement between two methods, and *K* > 0.41 and ≤ 0.61 show a moderate agreement between two methods [34].

## Results and discussion

### Characterization of ML-ES

**Fig. 2:**
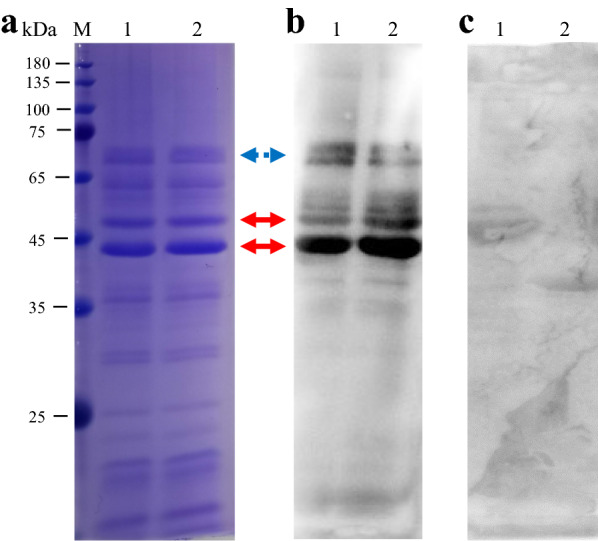
SDS-PAGE and WB identification of ML-ES. **a** ML-ES were characterized by SDS-PAGE. **b** ML-ES antigens were detected by *T. spiralis*-infected pig serum. **c** ML-ES antigens were detected by uninfected pig serum as negative control. These experiments included two repeats (1, 2). M, marker proteins

To verify the quality of the ML-ES preparations, SDS-PAGE was used to evaluate their protein size and composition, and the main proteins were nearly 45 kDa (Fig. 2a), which is consistent with a previous study [35]. WB revealed that the ML-ES reacted well with the positive serum in vitro (Fig. 2b) but not with the negative serum (Fig. 2c), and the main reactive proteins were nearly 45 kDa (Fig. 2b). Thus, the prepared ML-ES is a reliable candidate to detect anti-*T. spiralis* IgG antibodies in pig serum.

### Synthesis confirmation of UCNPs-ES and UCNPs-goat anti-rabbit IgG

**Fig. 3:**
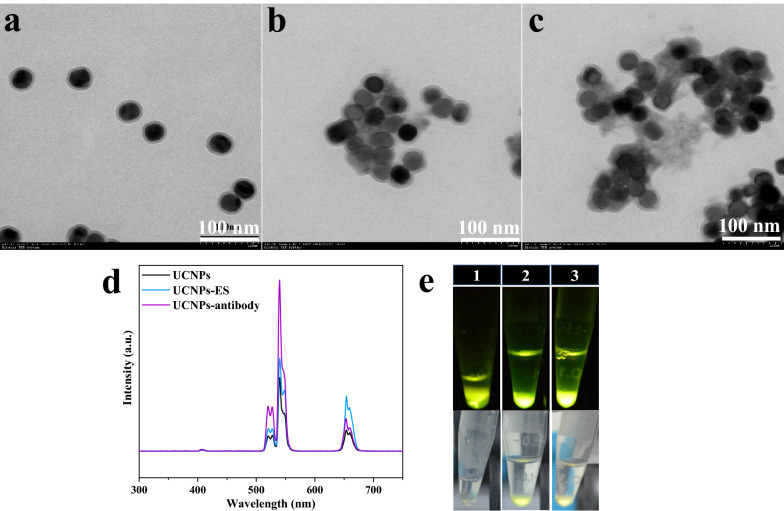
Characterization of UCNPs-ES/goat anti-rabbit IgG. TEM images of unconjugated UCNPs (**a**), UCNPs-ES (**b**), and UCNPs-goat anti-rabbit IgG (**c**) are shown. The spectra stimulated in 980 nm laser light are shown (**d**), and the physical pictures were also obtained in dark and light environments (**e**), including unconjugated UCNPs solution (1), UCNPs-ES solution (2), and UCNPs-goat anti-rabbit IgG solution (3)

To determine whether the UCNPs were coupled with the ES or goat anti-rabbit IgG, their morphology and size were evaluated by TEM to compare the change between unconjugated UCNPs and conjugated UCNPs. The results showed that the UCNPs maintained a well-dispersed character regardless of conjugation, but the rings around the conjugated UCNPs were enlarged compared with those of the unconjugated UCNPs as observed by TEM (Fig. 3a–c). Moreover, the diameter of the conjugated UCNPs included in the rings was enlarged overall compared with the unconjugated UCNPs by measuring the TEM images using software (Image-Pro Plus 6.0) (Additional file 1: Figure S1). In addition, the effects of fluorescence between the conjugated UCNPs and the unconjugated UCNPs were compared by using UC emission spectra and a 980 nm laser, and the results showed that the conjugation did not change the emission spectra and that the fluorescence intensity was related only to the concentration of UCNPs (Fig. 3d, e). These results indicated that ES/goat anti-rabbit IgG was successfully immobilized on the UCNPs.

### Optimization of the UPT-LF-ES assay and the optimized results

**Fig. 4:**
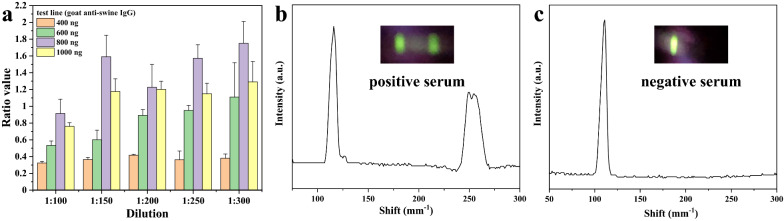
Optimization of the UPT-LF-ES assay and the optimized results. **a** The dosage of goat anti-swine IgG (400, 600, 800, 1000 ng/4 mm) in the T-line and the dilution of serum (1:100, 1:150, 1:200, 1:250, 1:300) were optimized to obtain better conditions (T-line: 800 ng of goat anti-swine IgG; dilution of serum: 1:150). Positive serum (**b**) and negative serum samples (**c**) were tested under the optimized conditions, and the inserted photographs showed the results under 980 nm laser light

To achieve an UPT-LF-ES assay with optimal performance, the dosage of goat anti-swine IgG in the T-line and the serum dilution for the samples were optimized. We comprehensively considered that the dosage of goat anti-swine IgG in the T-line should be 800 ng/strip (width: 4 mm), and the dilution of serum for samples should be 1:150 but not 1:300 (Fig. 4a). Due to the sensitivity of detection was reduced by the high level of dilution for the serum, and the performances are also similar between 1:150 and 1:300. Under the optimized conditions, Fig. 4b, c show the detection results from the UPT-based biosensor for a standard positive sample and negative sample from our lab, respectively, and the inserted photographs were obtained under 980 nm laser light. The results can easily identify positive or negative samples with 980 nm laser light regardless of whether a biosensor or the naked eye was used, which is satisfying.

### Cut-off threshold of the UPT-LF-ES assay

**Fig. 5:**
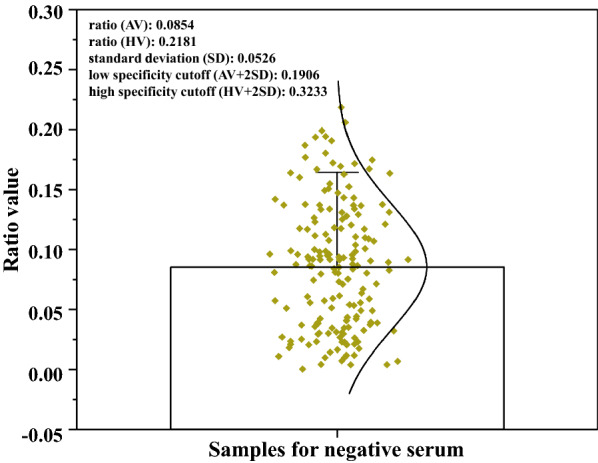
Cut-off threshold of the UPT-LF-ES assay. The cut-off thresholds were obtained by 169 known negative serum samples, including a low specificity cut-off (ratio: 0.1906) and a high specificity cut-off (ratio: 0.3233). AV, Average value; HA, Highest value

The ratio values obtained from these sera ranged from 0.0004 to 0.2181 (median, 0.0865), with an average of 0.0854 and a standard deviation (SD) of 0.0526 (Fig. 5). The cut-off threshold above which a sample was designated a “positive sample” was set to ≥ 0.3233 (highest negative sample value plus 2 SD), and the cut-off threshold below which a sample was designated a “negative sample” was set to ≤ 0.1906 (average plus 2 SD). Samples resulting in ratio values between 0.1906 and 0.3233 were designated “potentially positive sample”. The system of cut-off threshold is more scientific and has been used in the test for neurocysticercosis [26].

### Performance of the UPT-LF-ES assay to *T. spiralis* with different infective numbers

**Fig. 6:**
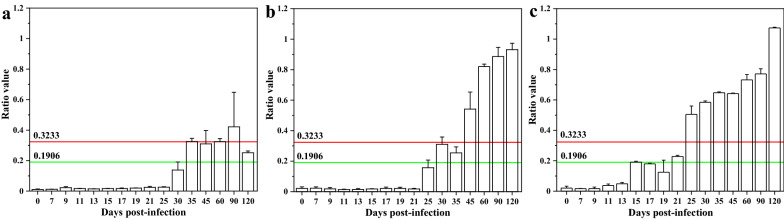
Performance of the *T. spiralis* assay with different infective numbers. Serum samples from pigs experimentally infected with 100 (**a**), 1000 (**b**), or 10,000 (**c**) *T. spiralis* were tested using this assay. The green line is the low specificity cut-off of 0.1906, and the red line is the high specificity cut-off of 0.3233. Data are presented as the mean ± SD from three independent experiments

According to the optimized procedure and the established cut-off threshold, a set of different *T. spiralis* infection doses were tested to preliminarily evaluate the performance of this assay. Figure 6 shows that positive results were first presented at 35 dpi, 30 dpi, and 25 dpi in the 100, 1000, and 10,000 ML infection groups, respectively. Additionally, the anti-*T spiralis* antibody in serum is positively correlated to the infection dose. More importantly, the first positive time point was impacted by the time of seroconversion [36], and the level of the anti-*T spiralis* IgG antibodies at a low infection dose (100 ML) was reduced at 120 dpi, which was related to the intensity of the first induced immune response in pigs. Overall, the assay is comparable to an indirect ELISA and a western blot assay at these infection doses [31, 37].

### The results from the sera of heterologous infection*s*

**Fig. 7:**
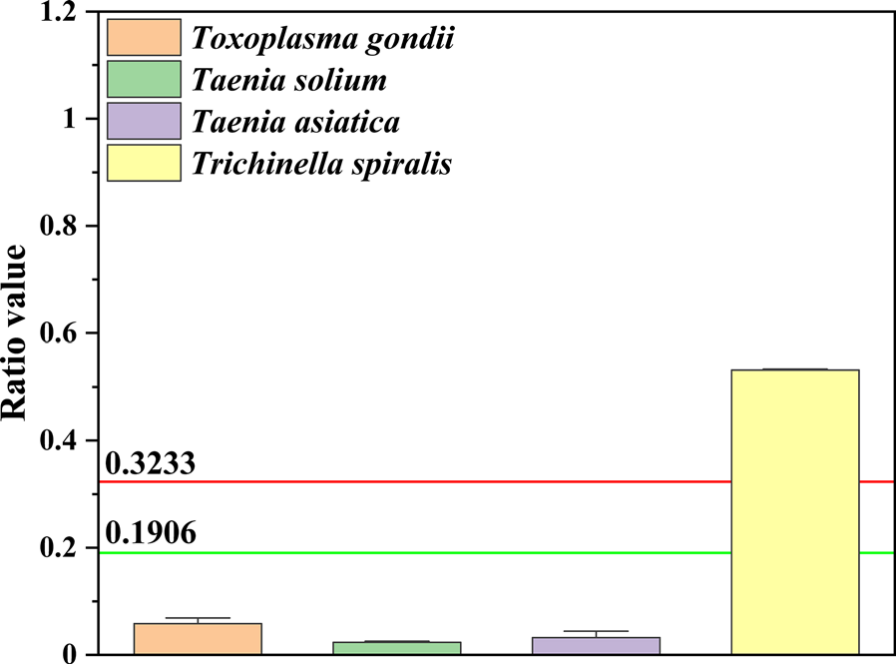
Cross-reactivity of the assay with sera infected with other parasites

To evaluate potential cross-reactivity and cut-off threshed of the assay, this assay was used to detect a set of serum samples from pig with other parasitic infections, including *Toxoplasma gondii*, cysticerci of *Taenia solium*, and cysticerci of *Taenia asiatica*. The ratio values from the *T. spiralis* test were significantly higher than those of the samples with other parasitic infections using this assay. The samples with other parasitic infections were also clearly low compared with the cut-off threshold of the “negative sample” (Fig. 7), and the results showed that the assay has no cross-reactivity with these tested other parasites serum samples, and the cut-off threshold is also acceptable.

### The single-blinded experimental validation results

**Fig. 8:**
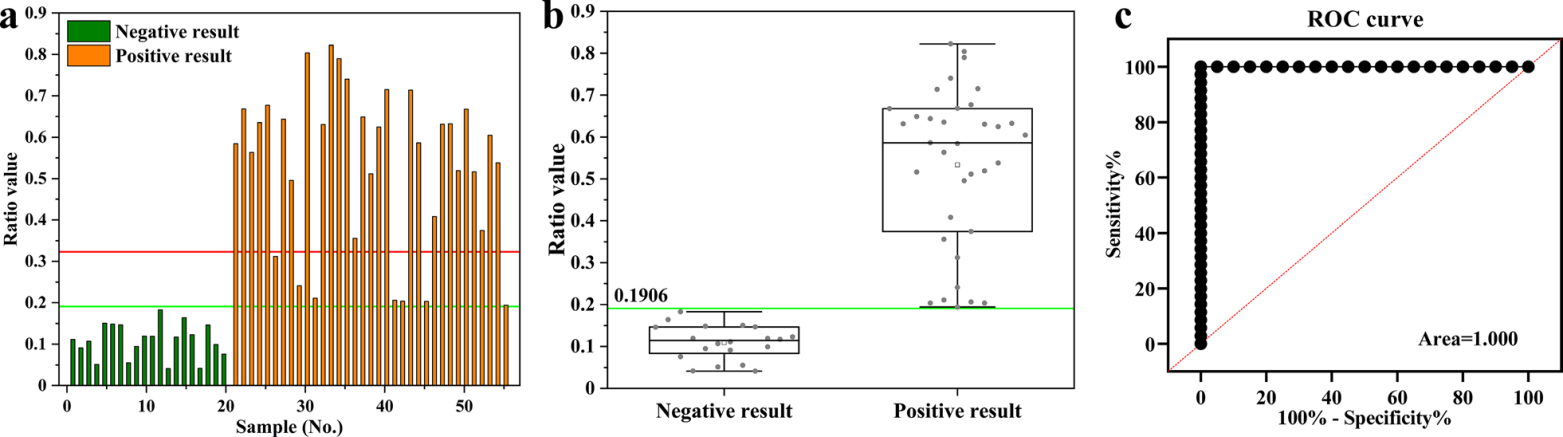
The results of single-blinded experimental validation. **a** The results of the 55 pig serum samples tested by the assay. **b** All of the results are expressed as the box plot. The ratio values of these two groups were significantly different (*t*_(53)_ = 9.827, *P* < 0.0001). Student’s *t*-test was used to determine significant differences. **c** ROC analysis of the 55 pig serum samples based on the ratio values of the assay. The integrated area under the ROC curve was 1.000

To analyze the effects of the UPT-LF-ES assay on the detection of *T. spiralis* infection in practice, a single-blinded experiment involving a total of 55 serum samples was employed. After the experiment, the results for each sample were presented and compared with two cut-off thresholds as shown in Fig. 8a. Based on the box plot of the results, the two groups were differentiated by the low specificity cut-off (0.1906). In addition, a *t*-test was used to evaluate whether there was a difference between the positive and negative samples, and the values were significant (*t*_(53)_ = 9.827, *P* < 0.0001) between the two groups (Fig. 8b), which indicated that the assay could effectively distinguish between the positive and negative samples by the number value. Moreover, we evaluated the performance with a ROC curve (Fig. 8c), and the area under the curve was 1.000, indicating that the assay is effective in distinguishing between positive and negative samples. According to this experiment, the sensitivity and specificity of the assay were 100% and 100%, respectively. Overall, the UPT-LF-ES assay showed good performance in the primary test.

### Comparative studies between the UPT-LF-ES assay and the magnetic stirrer method

The UPT-LF-ES assay and magnetic stirrer method, an artificial digestion technique considered to be the “gold standard” for the detection of *Trichinella*, were compared by statistical analysis, and the data are shown in Fig. 8a and our previous research [31]. Table 1 presents the details of the two assays; the total coincidence rate of the UPT-LF-ES assay was 87.3% (positive coincidence rate: 80%, negative coincidence rate: 100%) with substantial K value (*K* = 0.7454), indicating that the assay can be used to accurately detect anti-*T. spiralis* IgG antibodies in a pig serum sample. Due to the result of the cut-off system include the “potentially positive”, the total coincidence rate of the assay was not 100%, but this cut-off system is more scientific in a low prevalence of disease, such as *T. spiralis* infection, to effectively reduce false-positive results. In addition, this cut-off system has been adopted by the test of neurocysticercosis [26].

**Table 1:** The total coincidence rate of the magnetic stirrer method and the UPT-LF-ES assay

Group	Result	Magnetic stirrer method			Total
		Positive	Potentially positive	Negative	
UPT-LF-ES assay	Positive	28	0	0	28
	Potentially positive	7	0	0	7
	Negative	0	0	20	20
Total		35	0	20	55

Positive coincidence rate: 80%, negative coincidence rate: 100%, test coincidence rate: 87.27%

## Conclusions

In summary, this study developed a UCNPs-ES probe-based lateral flow assay for the rapid on-site detection of the anti-*T. spiralis* IgG antibody in pig serum to monitor the epidemic situation and ensure food safety. The UCNPs used in the LF assay have the advantages of a large anti-Stokes displacement, a long lifetime, tunable emission, high photostability, a sharp emission bandwidth, and low cytotoxicity. In addition, the UCNPs are excited in the near-infrared (NIR) spectral region. Compared with conventional ultraviolet (UV) and visible light, the NIR spectral region has the advantages of little autofluorescence, photodamage, and mutations in biological samples. Under the optimal conditions, the UPT-LF-ES assay was also integrated for the rapid, specific, and sensitive detection of *T. spiralis* infection using pig serum samples to prevent human infection by eating raw or undercooked pork. Compared with the “gold standard” magnetic stirrer method, the UPT-LF-ES assay has not only good agreement but also more convenient operation, which can be applicable for an on-site test.

## Supplementary Information

**Additional file 1: Figure S1.** The size of conjugated and unconjugated UCNPs was measured by software for TEM images. **a** Unconjugated UCNPs; **b** UCNPs-ES; **c** UCNPs-goat anti-rabbit IgG. **d** The statistical analysis of the size of unconjugated and conjugated UCNPs was operated. The results are shown as means ± SE. *P* < 0.01 (**) indicating a statistically significant difference compared to the control group (Unconjugated UCNPs). ns, no significance; SE, standard error

## Data Availability

The data supporting the conclusions of this article are provided within the article. The original datasets analyzed in the present study are available from the corresponding author upon request.

## Abbreviations

UCNPs: Upconverting phosphor nanoparticles
ES: Excretory-secretory
ML: Muscle larvae
LF: Lateral flow
ROC: Receiver operating characteristic
POCT: Point-of-care test
ICT: International Commission on Trichinellosis
OIE: World Organization for Animal Health
ELISA: Enzyme-linked immunosorbent assay
UPT: Upconverting phosphor technology
