# Supplementary material for: Upconverting phosphor technology-based lateral flow assay for the rapid and sensitive detection of anti-Trichinella spiralis IgG antibodies in pig serum
Source: Parasit Vectors. 2021 Sep 22;14:487. doi: 10.1186/s13071-021-04949-2 (PMC8456594; doi:10.1186/s13071-021-04949-2)

**Additional figure captions**

**Figure S1.** The size of conjugated and unconjugated UCNPs were measured by a software for TEM images. **a** Unconjugated UCNPs; **b** UCNPs-ES; **c** UCNPs-goat anti-rabbit IgG. **d** The statistical analysis of the size of unconjugated and conjugated UCNPs was operated. The results are shown as means ± SE. *P* < 0.01 (**) indicating a statistically significant difference compared to the control group (Unconjugated UCNPs). *Abbreviations*: ns, no significance; SE, standard error


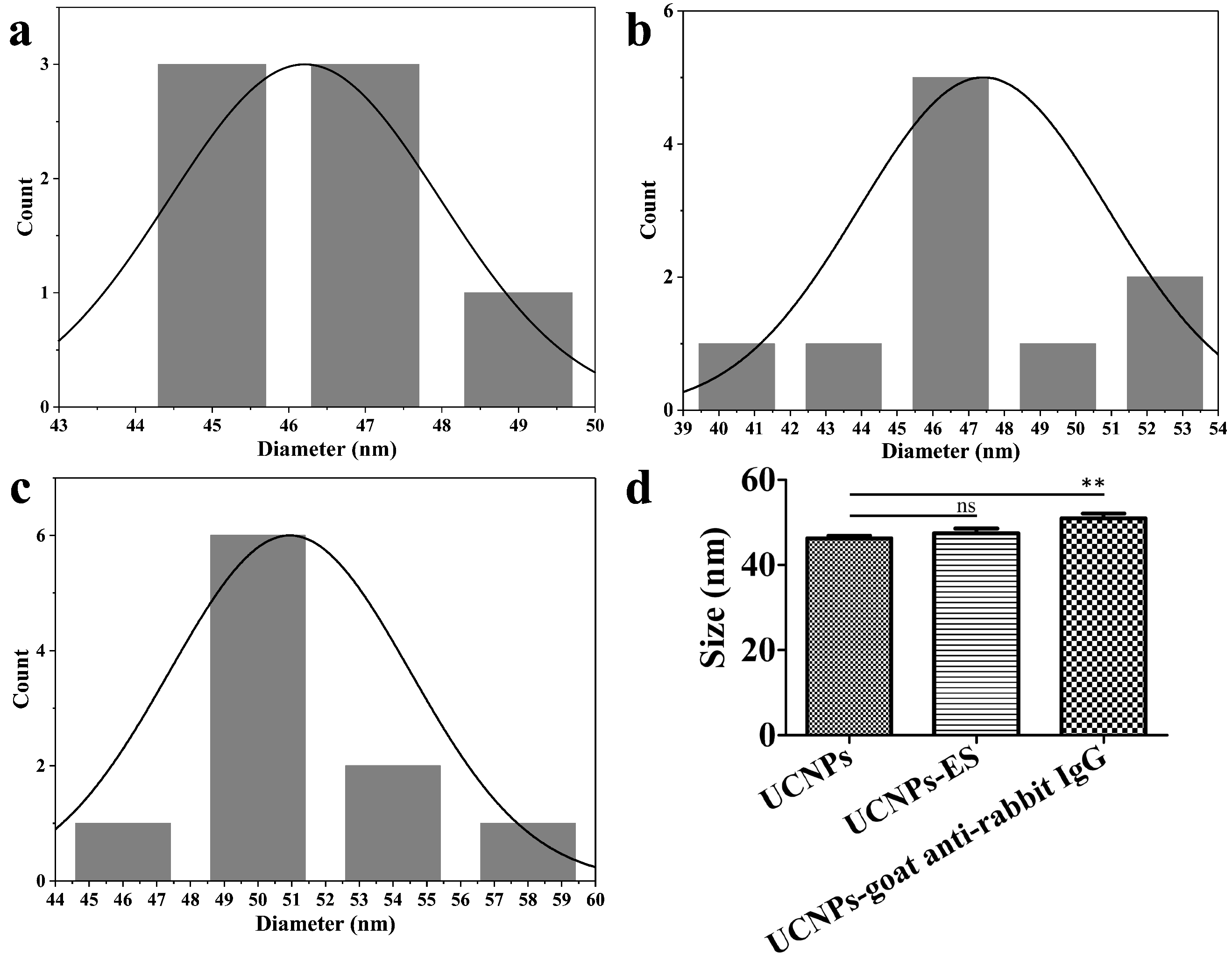

Supplement: Supplementary file 1 — Additional file 1: Figure S1. The size of conjugated and unconjugated UCNPs was measured by software for TEM images. a Unconjugated UCNPs; b UCNPs-ES; c UCNPs-goat anti-rabbit IgG. d The statistical analysis of the size of unconjugated and conjugated UCNPs was operated. The results are shown as means ± SE. P < 0.01 (**) indicating a statistically significant difference compared to the control group (Unconjugated UCNPs). ns, no significance; SE, standard error [file 13071_2021_4949_MOESM1_ESM.docx]
